# The Response Patterns of Arbuscular Mycorrhizal and Ectomycorrhizal Symbionts Under Elevated CO_2_: A Meta-Analysis

**DOI:** 10.3389/fmicb.2018.01248

**Published:** 2018-06-11

**Authors:** Yuling Dong, Zhenyu Wang, Hao Sun, Weichao Yang, Hui Xu

**Affiliations:** ^1^Key Laboratory of Pollution Ecology and Environmental Engineering, Institute of Applied Ecology, Chinese Academy of Sciences, Shenyang, China; ^2^University of Chinese Academy of Sciences, Beijing, China; ^3^School of Biological and Chemical Engineering, Liaoning Institute of Science and Technology, Benxi, China

**Keywords:** mycorrhizal fungi-plant symbiont, eCO_2_ fertilization effect, experimental duration, fertilization, global climate change

## Abstract

Elevated carbon dioxide (eCO_2_), a much-discussed topic in global warming, influences development and functions of mycorrhizal fungi and plants. However, due to the inconsistent results reported in various publications, the response patterns of symbionts associated with the arbuscular mycorrhizal (AM) or with ectomycorrhizal (ECM) fungi to eCO_2_ remains still unclear. Therefore, we performed a meta-analysis to identify how eCO_2_ affected mycorrhizal fungi and if there is a significant different response between AM and ECM symbionts. Our results demonstrated that eCO_2_ increased mycorrhizal plants biomass (+26.20%), nutrient contents [+2.45% in nitrogen (N), and +10.66% in phosphorus (P)] and mycorrhizal fungal growth (+22.87% in extraradical hyphal length and +21.77% in mycorrhizal fungal biomass), whereas plant nutrient concentrations decreased (−11.86% in N and −12.01% in P) because the increase in plant biomass was greater than that in nutrient content. The AM plants exhibited larger increases in their biomass (+33.90%) and in their N (+21.99%) and P contents (+19.48%) than did the ECM plants (+20.57% in biomass, −4.28% in N content and −13.35% in P content). However, ECM fungi demonstrated increased responses of mycorrhizal fungal biomass (+29.98%) under eCO_2_ compared with AM fungi (+6.61%). These data indicate different patterns in the growth of AM and ECM symbionts under eCO_2_: AM symbionts contributed more to plant growth, while ECM symbionts were more favorable to mycorrhizal fungal growth. In addition, the responses of plant biomass to eCO_2_ showed no significant difference between short-term and long-term groups, whereas a significant difference in the responses of mycorrhizal fungal growth was found between the two groups. The addition of N increased plant growth but decreased mycorrhizal fungal abundance, and P addition increased total plant biomass and extraradical hyphal length, but shoot biomass largely increased in low P conditions. Mixtures of mycorrhizal fungi affected the total plant and root biomasses more than a single mycorrhizal fungus. Clarifying the different patterns in AM and ECM symbionts under eCO_2_ would contribute to a better understanding of the interactions between mycorrhizal fungi and plant symbionts under the conditions of global climate change as well as of the coevolution of flora with Earth's environment.

## Introduction

The rising level of carbon dioxide (CO_2_) in the atmosphere is a major concern worldwide and could cause many changes in plant physiology and metabolism (Leakey et al., 2009; McGrath and Lobell, 2013). Individual studies have been conducted to assess the effects of elevated CO_2_ (eCO_2_) levels on plant growth, including nutrient absorption (Feng et al., 2015), the net assimilation rate, stomatal conductance (Augé et al., 2015), transpiration, water-use efficiency and sugar accumulation in leaves (Drake et al., 1997). It has been reported that eCO_2_ has changed the ecosystem element cycles, and many earth system models (ESMs) have been established to predict the future carbon (C), nitrogen (N), and phosphorus (P) cycles, as well as their interactions (Zaehle et al., 2014; Reed et al., 2015). In addition, excessive fertilizer input would further complicate the C-N-P interactions under eCO_2_ conditions. Moreover, mycorrhizal fungi, probably the most widespread symbionts in nature, exist in approximately 80% of terrestrial plant species (Baum et al., 2015). Extensive interactions exist between mycorrhizal fungi and plant symbionts: mycorrhizal fungi transfer N and P to plants, while plants supply organic carbon (C) to mycorrhizal fungi (Smith and Smith, 2012). This phenomenon improves plant growth, nutrient absorption, and water-use efficiency (Smith and Smith, 2012). By altering stomatal conductance, making osmotic adjustments (Augé et al., 2014) and regulating related gene expression (Porcel et al., 2016), among other mechanisms, the symbionts alleviate the harmful effects of drought and salinity stress on their plant hosts (Augé et al., 2015). Furthermore, mycorrhizal fungi can contribute to the alleviation of heavy metal pollution (Curaqueo et al., 2014; Yang et al., 2015), heat stress (Prasad et al., 2008; Cabral et al., 2016), ozone stress (Cui et al., 2013), and soil aggregation (Leifheit et al., 2014; Rillig et al., 2015). Therefore, discussing the responses of plants to eCO_2_, their interactions with mycorrhizal fungi must be considered (Grover et al., 2015; Simonin et al., 2017).

Arbuscular mycorrhizal (AM) and ectomycorrhizal (ECM) fungi employ two different nutrient acquisition strategies: AM fungi scavenge for nutrients released by saprotrophic microbes, whereas ECM fungi mineralize nutrients from organic matter and can thus access some forms of organic N directly (Phillips et al., 2013). It is therefore expected that AM and ECM might respond differently to eCO_2_ levels (Treseder and Allen, 2000; Alberton et al., 2005). However, inconsistent results of AM colonization were reported, including positive effects (Becklin et al., 2016; Jakobsen et al., 2016), negative effects (Goicoechea et al., 2014) and no effect (Tang et al., 2006), and similar inconsistent results were found for ECM (Walker et al., 1998; Garcia et al., 2008; Wang et al., 2015). Mycorrhizal fungal biomass has been reported to show positive or negative effects in different AM species (Langley et al., 2003), and this is also the case in ECM (Gutknecht et al., 2012). AM and ECM trees are also expected to respond differently to global change factors due to their different adaptations and distribution patterns (Phillips et al., 2013). In previous studies, it was reported that AM trees exhibited positive responses and ECM more often demonstrated negative responses (Boggs et al., 2005; Quinn Thomas et al., 2009). Additionally, the fungal community is tightly linked to fine root production in plants under eCO_2_ (Lipson et al., 2014), and the characteristics of the plant type and relevant physicochemical factors induced by eCO_2_ may be important key factors in structuring the response of the microbial community to environmental change (Lee et al., 2015). N and P additions reportedly affect the growth of both mycorrhizal fungi and plants under eCO_2_ (Staddon et al., 2004; Lee et al., 2015; Ekblad et al., 2016; Jakobsen et al., 2016). Overall, it is difficult to draw a consistent conclusion and determine the magnitude of the effect without a statistical analysis due to the above mentioned inconsistent results in individual studies. Meta-analysis is a quantitative statistical method that integrates the results of numerous individual studies and can be used to extract a general trend from numerous individual results in a precise statistical manner. Therefore, in recent studies, meta-analysis was widely used to assess the overall summary effect of variables.

Plant responses to eCO_2_ have been thoroughly researched through meta-analyses, and plant growth has been shown to increase to different extents under eCO_2_ (Curtis and Wang, 1998; Poorter and Pérez-Soba, 2001; Jablonski et al., 2002; Ainsworth and Long, 2005; De Graaff et al., 2006; Duval et al., 2012). Nevertheless, the interactions between mycorrhizal fungi and plants under eCO_2_ were not considered in these previous studies, and only a few meta-analyses have addressed mycorrhizal fungi under eCO_2_ (Treseder, 2004; Alberton et al., 2005; Terrer et al., 2016). Treseder (2004) showed a greater increase in AM abundance compared to ECM abundance under eCO_2_ but did not assess the difference between AM and ECM plant responses. Alberton et al. (2005) reported higher response in ECM growth compared to AM fungal growth and a slightly but not significantly higher response in ECM compared with AM plant growth using a mixed parameters method. Terrer et al. (2016) showed a higher increase in biomass in ECM plants than in AM plants under both N-limiting and non-N-limiting conditions. Since inconsistent patterns of AM and ECM plants symbionts were reported in current meta-analysis studies, it is difficult to reach a conclusion of how eCO_2_ affects mycorrhizal plants and fungal growth in AM and ECM symbionts, Therefore, we conducted a meta-analysis to determine the different effects of eCO_2_ on AM and ECM symbionts.

In this meta-analysis, the effect sizes of 27 individual variables (Table 1) were calculated from 434 observations from 1987 to 2016 to quantify the effects of the individual variables on mycorrhizal fungal and plant growth under eCO_2_. Additionally, the meta-analysis aimed to answer the following questions: (1) what are the different patterns of mycorrhizal plant biomass and nutrients and of mycorrhizal fungal growth in AM and ECM symbionts under eCO_2_? and (2) how do other factors (species richness, experimental duration and fertilization) affect mycorrhizal plant and fungal biomass, and do they exhibit any interesting patterns under eCO_2_? Determining the patterns of mycorrhizal plant and fungal growth under eCO_2_ would help to improve our understanding of the interactions between mycorrhizal plant and fungal symbionts during the current global climate change involving eCO_2_.

**Table 1:** Heterogeneity statistics and percentage change for the 27 summary effect sizes under eCO_2_.

**Variables**	**Q*t***	**df**	***P*_hetero_**	***I*^2^ (%)**	**Percentage change (%)**
Total plant biomass	289.76	198	0.000	31.67	26.20
Leaf or needle biomass or area	102.61	26	0.000	74.66	24.50
Shoot biomass	403.60	210	0.000	47.97	23.45
Root biomass	424.81	165	0.000	61.16	34.43
Shoot-to-root ratio	545.61	159	0.000	70.86	−8.84%
N content in total plant	50.35	19	0.000	62.27	2.45%
P content in total plant	14.15	12	0.291	15.20	10.66
N content in root	22.88	20	0.295	12.58	21.31
P content in root	6.69	17	0.987	0.00	46.31
N content in shoot	64.98	24	0.000	63.07	−9.62
P content in shoot	239.38	21	0.000	91.23	−6.39
N concentration in total plant	53.98	12	0.000	77.77	−11.86
P concentration in total plant	28.15	11	0.003	60.92	−12.00
N concentration in root	30.47	28	0.341	8.10	−7.14
P concentration in root	213.76	45	0.000	78.95	−3.34
N concentration in shoot	87.31	40	0.000	54.19	−25.10
P concentration in shoot	118.61	39	0.000	67.12	−10.08
N concentration in leaf	152.92	17	0.000	91.42	−15.17
P concentration in leaf	193.68	37	0.000	88.89	−12.01
Net photosynthesis assimilation rate	145.89	15	0.000	89.72	27.47
TSS concentration in leaf	82.48	16	0.000	80.60	26.67
Mycorrhizal fungal colonization	539.71	220	0.000	59.24	14.40
Root with hyphae	45.05	43	0.386	4.55	7.47
Root with arbuscules	180.52	52	0.000	71.19	31.00
Root with vesicles	15.63	14	0.337	10.43	36.48
Extraradical hyphal length	140.82	78	0.000	44.61	22.88
Mycorrhizal fungal biomass	34.19	44	0.856	0.00	21.77

*Qt, total heterogeneity; P_hetero_, probability that the observed heterogeneity was due to sampling error; I^2^, percentage of heterogeneity due to true variation among effect sizes; TSS, total soluble sugar*.

## Materials and methods

### Data collection

Publications were searched using the ISI Web of Science search tool (Thompson Reuters). On December 12, 2016, we conducted a search using the terms “mycorrhiz^*^” and “CO_2_” or “mycorrhiz^*^” and “carbon dioxide,” and the search resulted in 1,140 publications. Papers were included when they met the following criteria: mycorrhizal fungi can be clearly identified as AM or ECM; at least one of the 27 variables was given; and means and sample sizes were reported. For each paper, data resulting from studies with different mycorrhizal fungal species, host plant species, experimental durations and nutrients levels were considered independent studies. When multifactorial studies appeared, only data of control groups and eCO_2_ groups were used. When papers reported the same data, we selected one of them (Kohler et al., 2009, 2010). We obtained 434 observations from 112 papers (Appendix S1) by using the above criteria and removing the duplicates.

The mycorrhizal fungal and plant species richness, experimental durations, fertilization conditions (**Figure 2**) and 27 variables (Table 1) were collected for each study. Engauge software was used to extract data that were provided in graphical form. When observations lacked total plant biomass, shoot biomass, root biomass or shoot-to-root ratio, the missing parameters were calculated using the following formulas: total biomass = shoot biomass + root biomass and shoot-to-root ratio = shoot biomass/root biomass. Standard deviations (SDs) were calculated when only standard errors (SEs) were reported by using the equation SD = SE × sqrt(n). Unidentified error bars were assumed to represent SEs. For the studies that did not report SDs, we calculated the average coefficient of variation (CV) within each dataset and then approximated the missing SD by multiplying the reported mean by the average CV. The number of treatments listed in the text was replicates of a treatment rather than the sample size per treatment. When the value of n was given as a range, the smallest value was taken.

### Moderators

Each mycorrhizal fungal type was grouped into either the AM or the ECM category. Plant and mycorrhizal fungal species richness was grouped into the “single” and “mixture” categories. Treatment durations spanned from 5 days to 14 years and were grouped into two experimental durations: ≤1 year and >1 year. The fertilization conditions included two groups: N addition (high N and low N) and P addition (high P and low P). The ambient CO_2_ levels ranged from 336 to 400 ppm, and the eCO_2_ levels ranged from 550 to 1,000 ppm except in four studies that reported eCO_2_ concentrations greater than 1,000 ppm (1,500, 3,360, and 10,000 ppm in two studies), which were included in our meta-analysis.

All 27 variables (Table 1) except the mycorrhizal fungal biomass were used individually in the meta-analysis according to Augé et al. (2014). Alberton et al. (2005) tested whether mycorrhizal plants and mycorrhizal fungi responded similarly under eCO_2_. To extract as much information as possible from the scarce papers available at the time, Alberton et al. provided an order of measurements to ensure that the plant and fungal responses were maximally different and noted the possibility that not all parameters were unbiased. The meta-analysis of mycorrhizal fungal biomass in our study was conducted according to the method described by Alberton et al. (2005) because there were not enough observations representing the allocation of mycorrhizal fungal biomass.

The data used for calculating the mycorrhizal fungal biomass responses included the dry weight of extraradical mycelia, extramatrical hyphae mass, hyphal biomass, fungal biomass in soil, total fungal biomass, ECM tip biomass, mycorrhizal mass, fungal biomass in root, specific phospholipid fatty acid (PLFA) content, neutral lipid fatty acid (NLFA) content, and ergosterol content according to the rank-order method described by Alberton et al. (2005).

### Meta-analysis

The response ratio (*R*), which was defined as the “effect size,” was calculated as the ratio of the values in the eCO_2_ treatment group (*X*_*t*_) to those in the control group (*X*_*c*_) (Hedges et al., 1999). We performed a log transformation on the response ratio *R* to develop a better statistical understanding as follows (Hedges et al., 1999):

(1) $$\begin{array}{l} {\text{log}}_{e}\;R={\text{log}}_{e}\;\left(\frac{X_{t}}{X_{c}}\right)={\text{log}}_{e}\;\left(X_{t}\right)-{\text{log}}_{e}\;\left(X_{c}\right) \end{array}$$

The variance of log_e_ *R* was calculated using the following formula:

(2) $$\begin{array}{l} v=\frac{s_{t}^{2}}{ntX_{t}^{2}}+\frac{s_{c}^{2}}{ncX_{c}^{2}} \end{array}$$

where *s*_*t*_ and *s*_*c*_ represent the SDs of the treatment and control groups, respectively. In addition, *n*_*t*_ and *n*_*c*_ are the sample sizes of the treatment and control groups, respectively.

The variance, *v*_*n*_, was adjusted by the number of observations (*n*) in each study and was calculated by the following formula:

(3) $$\begin{array}{l} v_{n}=v\times n \end{array}$$

where *n* represents the number of observations from the same publication.

The final weighted effect sizes, log_*e*_ *R*_*i*_′, and the mean effect sizes, were calculated by MetaWin 2.0 software using the log_*e*_ *R*_*i*_′ and *v*_*n*_ values.

To obtain a clearer understanding, the percentage changes of the mean effect sizes were transformed with the following formula:

(4) $$\begin{array}{l} \left(e^{\bar{log_{e}R^{\prime }}}-1\right)\times 100\% \end{array}$$

These four equations were described in Bai et al. (2013).

Heterogeneity was estimated with the *Q* statistic in MetaWin 2.0 software. Total heterogeneity (*Q*_*t*_) was composed of the difference between group cumulative effect sizes (*Q*_*m*_) and residual error (*Q*_*e*_) (Rosenberg et al., 2000). *I*^2^ is an index that assesses the ratio of true heterogeneity to the total heterogeneity across the observed mean effect sizes and is calculated as: (*Q*_*t*_ – *df*)/*Q*_*t*_, where the degrees of freedom (*df*) represent the expected variation and (*Q*_*t*_ – *df*) represents the true heterogeneity (Rosenberg et al., 2000; Higgins and Thompson, 2002; Huedo-Medina et al., 2006; Borenstein et al., 2009). The value of *I*^2^ ranges from 0 to 100%. The value of 0% indicates no heterogeneity exists among the variable dataset. The larger the value of *I*^2^, the larger the dataset's true heterogeneity. Some studies suggested that assumptions of heterogeneity were considered invalid when *p*-values were less than 0.1 (Higgins et al., 2003; Wilson et al., 2005; Allen et al., 2008). In this study, we used a random-effect model for all of the variables in the meta-analysis. When significant heterogeneities were identified among studies, the sources of true heterogeneity were investigated with moderator or subgroup analysis.

The 95% confidence intervals (CIs) of the weighted effect sizes were obtained using the bootstrapping (9,999 iterations) function in MetaWin software. The weighted mean effect size of a variable was considered significant when the 95% CI did not overlap zero and the *p*-value was less than 0.05 (Borenstein et al., 2009). A random-effect model was used to test the relationships between the weighted mean effect sizes of all the variables, experimental duration and the CO_2_ fold change using MetaWin software.

Publication bias was estimated with the Egger test function in Stata 12.0 software, and the estimates were obtained from the mean effects and variance. A *p*-value < 0.05 indicated a potential publication bias was present (Egger et al., 1997; Sterne et al., 2000; Deeks et al., 2005). The trim and fill method was used when there was a publication bias in the variable dataset (Duval and Tweedie, 2000; Peters et al., 2007).

## Results

### Overall summary effects

A total of 112 papers were included in this meta-analysis. Egger test was conducted to assess publication bias on the mean effect sizes of the 27 variables mentioned in the Data Collection section of Materials and Methods. Seventeen of the 27 variables exhibited no publication bias (*p* > 0.05) in their mean effect sizes. The datasets of 10 variables showed potential publication biases (*p* < 0.05), but seven of these sets showed no publication bias with the unchanged mean effect sizes after a “trim and fill” correction (Table S1).

**Figure 1:**
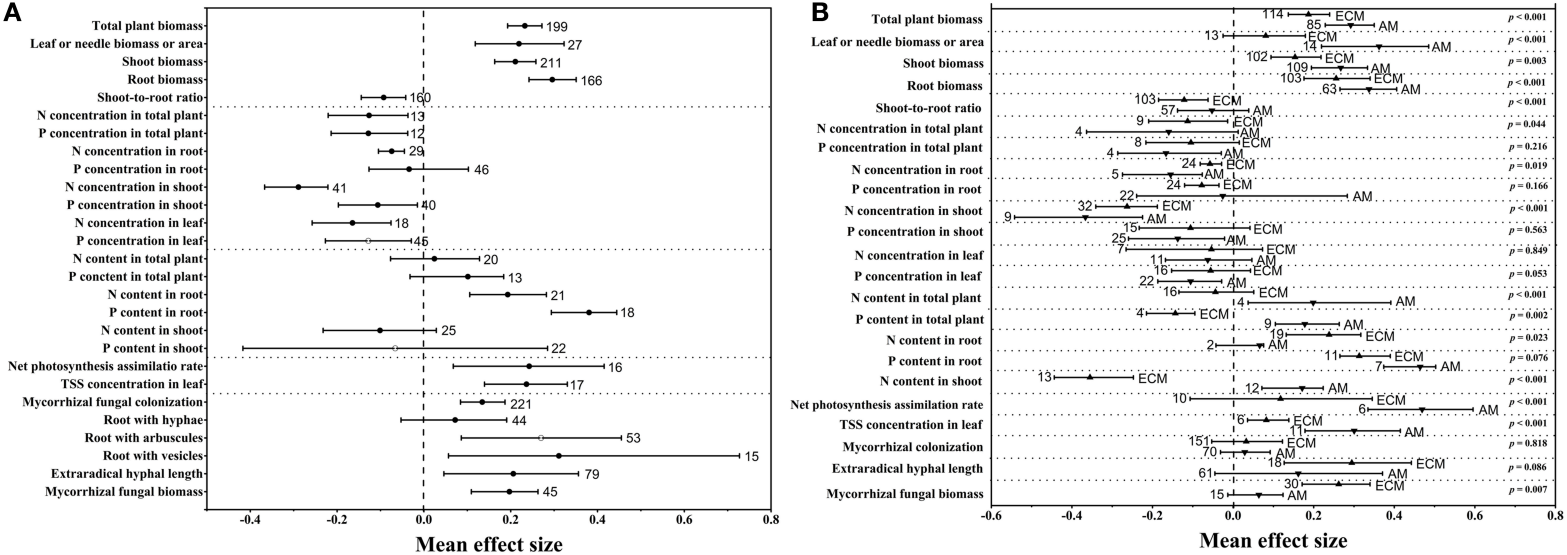
Mean effect sizes of eCO_2_ on 27 variables related to mycorrhizal fungi and plants in all groups **(A)** and in the AM and ECM **(B)** groups. TSS, total soluble sugar. Error bars represent 95% CIs. Open points are effect sizes that were corrected by the trim and fill method. The dashed line shows where the mean effect sizes are equal to zero. The effect size of eCO_2_ was considered significant when the 95% CI of the effect size did not contain zero. The sample size for each variable is shown next to the corresponding point.

The raw mean effect sizes of two variables (root with arbuscules and P concentration in the leaf) with true publication bias were slightly overestimated, but no subversive changes were found, and corrections were performed with the trim and fill method (Figure 1). Only the mean effect size of the P content in shoot, which was +0.126 and −0.066 before and after (Figure 1A) the adjustment, respectively, was changed subversively after trim and fill corrections.

As illustrated in Figure 1A, the mycorrhizal plant biomass responded positively to rising CO_2_ concentrations. The mean effect size of the total plant biomass was +0.233. The mean effect sizes of the leaf or needle biomass or area, shoot biomass and root biomass were +0.219, +0.211, and +0.296, respectively. However, there was a bias in the allocation of total biomass to the shoots and roots under eCO_2_, which led to a shoot-to-root ratio mean effect size of −0.093 (Figure 1A). Their 95% CIs did not overlap 0, indicating a significantly positive effect of the eCO_2_ atmosphere on plant biomass. Regarding mycorrhizal fungal development, CO_2_ enrichment significantly and positively affected extraradical hyphal length, with a mean effect size of +0.206 (Figure 1A). However, there was no significant difference in extraradical hyphal length between AM and ECM fungi (Figure 1B), although eCO_2_ significantly affected ECM extraradical hyphal length but had an insignificant effect on AM fungi. The mycorrhizal fungal biomass increased by +21.77% (Figure 1A), which was significant. Mycorrhizal fungal colonization, a variable associated with plants and mycorrhizal fungi, was significantly increased under eCO_2_. Moreover, the mean effect sizes of eCO_2_ on roots with hyphae, arbuscules and vesicles were +0.072, +0.270, and +0.311, respectively (Figure 1A). The AM plants displayed significantly larger effect sizes on biomass and nutrient contents than the ECM plants (Figure 1B). However, the ECM fungi showed a greater mycorrhizal fungal biomass response than the AM fungi (Figure 1B). The N and P concentrations were significantly decreased in all organs under eCO_2_ except for the P concentrations in root (Figure 1A). CO_2_ enrichment had a significantly positive effect on the N and P contents in root, whereas nonsignificant effects were found on the total plant and shoot N and P contents (Figure 1A). The N (*p* < 0.001) and P contents (*p* = 0.002) showed greater responses in whole AM plants than in whole ECM plants (Figure 1B). Significantly positive effects of eCO_2_ on the net photosynthesis assimilation rate (P_n_) and total soluble sugar (TSS) concentration in the leaf were also found in this study (Figure 1A), and both responses were increased more in AM plants than in ECM plants (Figure 1B).

### Subgroup moderator analysis

To test for interesting patterns, a subgroup analysis was conducted with six variables (total plant biomass, shoot biomass, root biomass, mycorrhizal fungal colonization, extraradical hyphal length, and mycorrhizal fungal biomass) that directly reflect the mycorrhizal fungi and plant growth and five moderators (plant species richness, mycorrhizal fungal species richness, experimental duration, N addition, and P addition).

### Species richness of mycorrhizal fungi and plants

**Figure 2:**
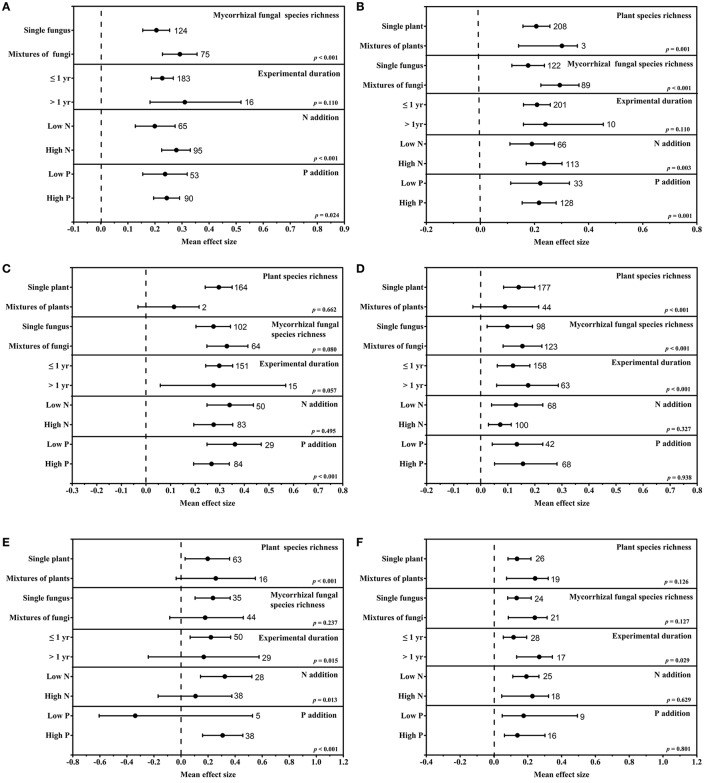
Subgroup moderator analysis of the mean effect sizes of eCO_2_ on the total plant biomass **(A)**, shoot biomass **(B)**, root biomass **(C)**, mycorrhizal colonization **(D)**, extraradical hyphal length **(E)** and mycorrhizal fungal biomass **(F)**. Variables were subgrouped into the following moderators: species richness (plants and mycorrhizal fungi), experimental durations and fertilization conditions (N addition and P addition). Missing subgroup moderators are absent due to inadequate observations involving those moderators in the meta-analysis. The dashed line shows where the mean effect size is equal to zero. The effect size of eCO_2_ was considered significant when the 95% CI of the effect size did not contain zero. The sample size for each moderator is shown next to the corresponding point.

Species richness affected the responses of mycorrhizal fungi and plants differently. Specifically, the plant species richness significantly affected the shoot biomass (*p* = 0.001), mycorrhizal fungal colonization (*p* < 0.001) and extraradical hyphal length (*p* < 0.001), whereas the mycorrhizal fungal species richness significantly influenced the total plant biomass (*p* < 0.001), shoot biomass (*p* < 0.001) and fungal colonization (*p* < 0.001). The responses of mycorrhizal fungal colonization (Figure 2D) and extraradical hyphal length (Figure 2E) were greater in single plant than in mixtures of plants. The total plant biomass (Figure 2A), shoot biomass (Figure 2B) and mycorrhizal fungal colonization (Figure 2D) showed greater increases in mixtures of fungi than in single fungus.

### Experimental durations

The experimental duration significantly affected the mycorrhizal fungi but had no significant effects on mycorrhiza-associated plants. The extraradical hyphal length was more affected under short-term conditions than under long-term conditions, whereas the colonization and fungal biomass exhibited greater effects under long-term conditions (Figures 2D,E).

### Fertilization conditions

The addition of fertilizers (N or P) to the soil changed the mycorrhizal fungal and plant responses to eCO_2_. Plant biomass was enhanced more under high-nutrient conditions (Figure 2A). P addition significantly increased the shoot biomass, whereas N addition did not significantly affect the shoot biomass (Figures 2B,C). There was no significant difference in effect the on mycorrhizal fungal colonization and fungal biomass under eCO_2_ between N enriched and low N conditions (Figures 2D,F). The extraradical hyphal length showed a greater response under low N or high P conditions than under other conditions (Figure 2E).

The sources of true heterogeneity were calculated using the Q statistic method. Two of the 27 parameters in our study had an *I*^2^-value of zero, whereas the other 25 parameters had positive *I*^2^ values. Twenty of these 25 parameters had significant heterogeneity (*P*_hetero_ < 0.1) (Table 1), which indicated true variation among these results; random-effect models were then used to assess the final mean effect sizes. True heterogeneity indicates that certain moderators significantly influence the variables' responses to eCO_2_. Therefore, a subgroup analysis was conducted to investigate the sources of true heterogeneity in datasets of only five variables (Figures 2A–E) due to the limited number of observations. As illustrated in Figure 2, the plant species richness significantly affected the responses of shoot biomass, mycorrhizal fungal colonization, and extraradical hyphal length to eCO_2_. The mycorrhizal fungal species richness and P addition significantly affected the responses of all five variables except the extraradical hyphal length to eCO_2_. The experimental duration significantly affected the responses of mycorrhizal fungal colonization and extraradical hyphal length to eCO_2_. In addition, N addition significantly affected the responses of total plant biomass, mycorrhizal fungal colonization and extraradical hyphal length to eCO_2_.

### Meta-regression analysis

The mycorrhizal fungal biomass was significantly correlated with the experimental duration (Table 2). It increased with increasing experimental duration but exhibited a relatively small slope (+0.0032). The N and P concentrations in shoot and the N and P concentrations in root were significantly correlated with the CO_2_ fold change. The N (slope of approximately −0.063) and P concentrations in shoot (slope of −0.083) and the N (slope of −0.090) and P concentrations in root (slope of −0.075) decreased as the CO_2_ fold change increased. The extraradical hyphal length was significantly correlated with the CO_2_ fold change, with a slope of +1.100.

**Table 2:** Relationships between the effect sizes of eCO_2_ on mycorrhizal fungal and plant development parameters and the experimental duration and CO_2_ fold change.

	**Q*t***	**Q*m***	**Q*e***	**Slope**	***p*-value**	**Sample size**
**DURATION**						
Total plant biomass	156.11	0.58	155.53	0.0008	0.447	199
Leaf or needle biomass or area	17.71	0.43	17.28	−0.0058	0.514	27
Shoot biomass	109.70	0.002	109.70	−0.0002	0.969	211
Root biomass	162.78	0.10	162.68	−0.0009	0.753	166
Shoot-to-root ratio	100.00	0.05	99.95	−0.001	0.820	160
N content in total plant	16.14	0.02	16.13	−0.0018	0.897	20
P content in total plant	10.03	0.05	9.98	0.0145	0.819	13
N content in root	17.33	0.03	17.30	−0.0003	0.861	21
P content in root	6.69	0.95	5.74	−0.0078	0.329	18
N content in shoot	22.02	1.27	20.75	0.0251	0.260	25
P content in shoot	13.98	5.50	8.48	−0.1473	0.019	16
N concentration in total plant	8.45	1.90	6.55	0.0121	0.168	13
P concentration in total plant	13.33	3.62	9.71	0.0195	0.057	12
N concentration in root	27.91	0.05	27.86	−0.0025	0.830	29
P concentration in root	66.10	0.02	66.08	−0.0013	0.886	46
N concentration in shoot	24.58	0.12	24.46	0.0101	0.732	41
P concentration in shoot	29.50	1.97	27.53	0.0191	0.160	40
N concentration in leaf	9.03	1.59	7.44	0.0114	0.208	18
P concentration in leaf	20.91	0.25	20.65	−0.0017	0.617	38
Net photosynthesis assimilation rate	17.04	1.01	16.03	0.0065	0.314	16
TSS concentration in leaf	15.48	0.25	15.23	−0.0452	0.620	17
Mycorrhizal fungal colonization	143.34	0.195	143.15	−0.0004	0.659	221
Root with hyphae	45.05	5.38	39.67	0.0032	0.020	44
Root with arbuscules	34.53	1.37	33.17	0.0031	0.242	51
Root with vesicles	14.80	2.01	12.79	0.0064	0.156	15
Extraradical hyphal length	43.34	0.01	49.33	0.0002	0.908	79
Mycorrhizal fungal biomass	34.19	5.18	29.01	0.0032	0.023	45
**CO_2_ FOLD CHANGE**						
Total plant biomass	150.64	1.09	149.55	0.0147	0.296	188
Leaf or needle biomass or area	16.51	0.19	16.33	0.2064	0.666	24
Shoot biomass	102.43	0.02	102.41	0.0016	0.879	198
Root biomass	154.45	0.05	154.40	0.0023	0.817	154
Shoot-to-root ratio	87.35	0.35	86.99	−0.0068	0.553	149
N content in total plant	15.87	0.07	15.80	−0.0076	0.791	19
P content in total plant	11.16	0.47	10.69	0.0263	0.492	13
N content in root	16.16	0.99	15.17	−0.0297	0.321	19
P content in root	6.69	0.38	6.31	0.0254	0.537	18
N content in shoot	17.73	0.14	17.59	0.0141	0.708	23
P content in shoot	6.60	0.16	6.44	−0.0258	0.693	16
N concentration in total plant	9.04	0.04	9.01	0.0852	0.847	13
P concentration in total plant	11.17	0.22	10.95	0.1766	0.642	12
N concentration in root	30.46	10.22	20.24	−0.0896	0.001	28
P concentration in root	53.69	16.01	37.67	−0.0749	0.000	42
N concentration in shoot	81.56	46.22	35.34	−0.0631	0.000	40
P concentration in shoot	46.73	17.53	29.20	−0.0826	0.000	36
N concentration in leaf	9.71	0.41	9.30	0.3194	0.524	18
P concentration in leaf	27.20	1.75	25.46	0.2673	0.186	38
Net photosynthesis assimilation rate	14.26	0.45	13.81	0.1305	0.501	15
TSS concentration in leaf	23.05	3.62	19.43	0.7570	0.057	17
Mycorrhizal fungal colonization	136.27	0.18	136.09	0.0036	0.671	180
Root with hyphae	11.97	1.14	10.82	−1.1315	0.285	14
Root with arbuscules	14.67	0.33	14.34	0.4291	0.567	22
Root with vesicles	15.63	9.03	6.60	−2.3062	0.003	15
Extraradical hyphal length	97.43	22.87	74.57	1.0995	0.000	77
Mycorrhizal fungal biomass	21.45	1.93	19.52	−0.308	0.164	28

*Qt, total heterogeneity in effect sizes among studies; Qm, difference among group cumulative effect sizes; Qe, residual error; TSS, total soluble sugar. The relationship is significant when p < 0.05*.

## Discussion

A meta-analysis, which is a statistical analysis method that combines data from independent studies, is used to determine whether the effect of a variable is consistent across a dataset and to check the potential variance in the effects within the dataset (Huque, 1988; Gurevitch and Hedges, 2001; Borenstein et al., 2009). Biases, including both publication and research bias, are important and complicating issues in a meta-analysis. Publication bias commonly results in overestimated mean effect sizes and significance (Gurevitch and Hedges, 2001) and is generally caused when studies with negative results are less frequently accepted than studies with positive results. The trim and fill method is used to correct the publication bias in a meta-analysis (Duval and Tweedie, 2000). In our study, the publication bias among the 27 variable datasets was taken into consideration, and the mean effect sizes with publication bias were corrected using the trim and fill method according to the study of Duval and Tweedie (2000) (Table 1, Figure 1A). Research bias is a more troublesome issue in meta-analysis since it originates from a variety of sources (Gurevitch and Hedges, 1999). For instance, the objects of the studies, the methods and the experimental conditions are subjectively selected by researchers, potentially resulting in research bias. The selection of the parameters to investigate could also result in research bias. For example, fractional colonization was not the most suitable parameter for assessing the performance of mycorrhizal fungi under eCO_2_ but was used frequently in previous studies because it is relatively easy to assess (Alberton et al., 2005). Klironomos et al. (2005) demonstrated that the magnitude of changes in fungal species richness and function was smaller in response to gradually increasing CO_2_ than in response to abruptly rising CO_2_, indicating a possible overestimation of the effects of enriched CO_2_ in studies using abruptly rising atmospheric CO_2_. Until now, however, quantifying research bias has been difficult because no formula or model could be fitted to predict research bias.

Positive effects were found in the responses of mycorrhizal plants and fungi to eCO_2_ in our study. The increases in total plant biomass (+26.20%) was consistent with the increases observed in other meta-analyses [+28.8% in the study conducted by Curtis and Wang (1998), +47% in that performed by Poorter and Pérez-Soba (2001), and +31% in the analysis conducted by Jablonski et al. (2002)]. These above previous studies did not consider the mycorrhizal fungal interaction, whereas our study only included plants associated with mycorrhizal fungi. In addition, in our study, the increases in mycorrhizal fungal colonization length and mycorrhizal fungal biomass (+14.88 and +22.23%, respectively) were smaller than the increases in mycorrhizal fungal abundance and colonization (+47 and +36%, respectively) reported by Treseder (2004). This difference may be because the meta-analysis of Treseder (2004) included only field studies and the smaller number of observations (14) compared to the number of observations in our study (434). As shown in Figure 1A, eCO_2_ increased the plant biomass and led to a smaller shoot-to-root ratio, resulting in a larger increase in root biomass than shoot biomass under eCO_2_. The shoot-to-root ratio depends on the partitioning of photosynthesis products, which might be affected by environmental and nutrient conditions (Rogers et al., 1995). Previous studies have elucidated that the partitioning of dry matter into shoots and roots is determined by the internal balance between labile N and C in the shoot and root systems (Ericsson, 1995). The N:C ratios in the mycorrhizal plant shoots and roots were found to be decreased in our study. An overall negative effect (−0.144) on the root-to-shoot ratios of AM-colonized plants was detected in the meta-analysis conducted by Veresoglou et al. (2012), a result opposite to the effect on the shoot-to-root ratio observed in our study (−0.052). Moreover, factors other than the nutrient supply rates that decrease growth rates have been reported to increase the N:C and P:C ratios (Sterner et al., 2002). Decreased N:C and P:C ratios were found in our study. Various mechanisms that could explain the decreased N and P concentrations in plants under eCO_2_ include the following: (1) the N or P content of plant organs is diluted by the enhanced photosynthetic assimilation of C (Kuehny et al., 1991; Gifford et al., 2000) and secondary compounds (Gifford et al., 2000); (2) decreased transpiration leads to decreased N uptake (McDonald et al., 2002; Del Pozo et al., 2007); and (3) N is incrementally lost (Pang et al., 2006) and the mycorrhization status decreases (BassiriRad et al., 2001; Alberton et al., 2005). The decreased N:C and P:C ratios observed in our study may be explained by the first mechanism described above, i.e., that the enhanced photosynthetic assimilation of C dilutes the N and P contents in plants. Plants generally maintain a homeostatic N:P ratio that is sensitive to environmental changes (Loladze and Elser, 2011). However, significant different responses detected in mycorrhizal plants and mycorrhizal fungi (Alberton et al., 2005), which may result in more complicating changes in roots under eCO_2_ since the root is a dual organ. We further deduced that the different responses in mycorrhizal fungi and plants may result in a more imbalanced N:P ratio in roots. Our study demonstrated that the increase in the P content (+13.48%) under eCO_2_ was greater than that in the N content (+2.45%) and also found a +26.20% increase in total plant biomass. The enhanced total plant biomass and lower N:P ratio observed in our study could be explained by the growth rate hypothesis, which assumes a low plant N:P ratio when the growth rates are enhanced (Sterner et al., 2002; Elser et al., 2010). In addition, the meta-regression results demonstrated that the decreased N and P concentrations in shoots and roots were significantly correlated with the amount of CO_2_ level change. This correlation indicated that the N and P concentrations in plant organs were influenced more strongly by the CO_2_ concentration than by the exposure period (Table 2).

**Figure 3:**
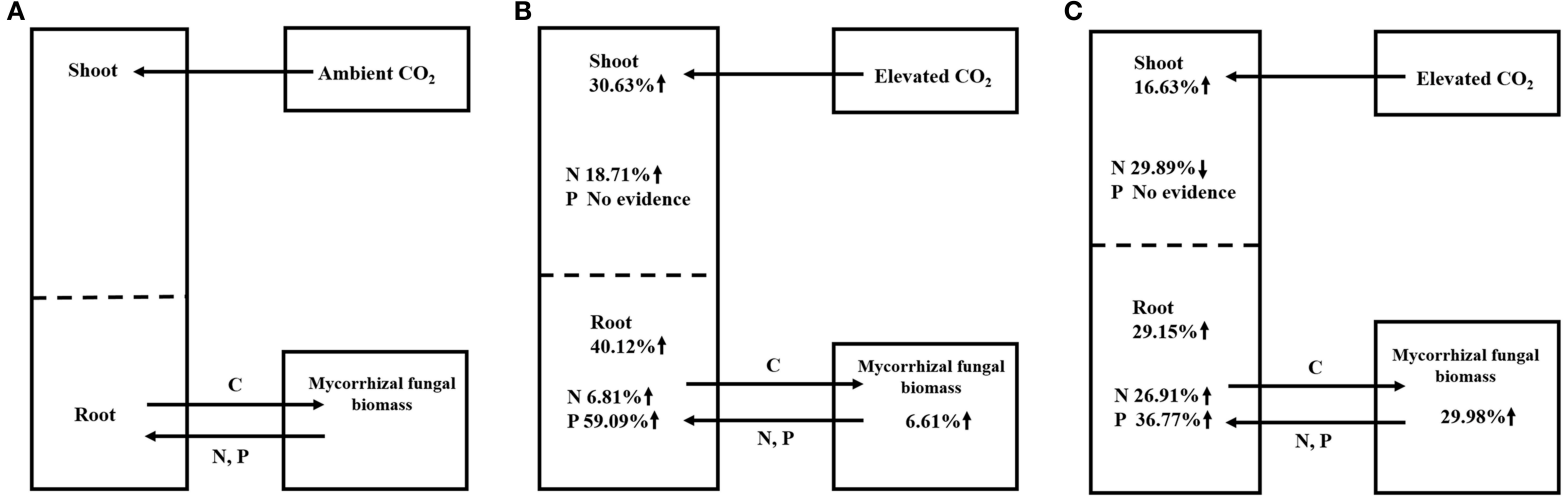
Summary of mycorrhizal plant-fungal symbiont responses to aCO_2_ **(A)**, AM symbiont responses to eCO_2_ **(B)**, and ECM symbiont responses to eCO_2_ **(C)**.

Previous studies showed no significant difference in the responses of ECM plants (+1.26) and AM plants (+1.25) (Alberton et al., 2005) or showed a larger effect in ECM plants (+33 ± 4%) than in AM plants (+20 ± 6%) (Terrer et al., 2016). However, our study demonstrated that AM plants showed larger increases in their biomasses (+33.90%) and their N (+21.99%) and P contents (+19.48%) than the ECM plants (changes of +20.57% in biomass, −4.28% in N content and −13.35% in P content). The above data indicating opposite results in our study compared to previous studies, and this may be explained by the different parameters used and different number of observations in these meta-analyses. For instance, Alberton et al. (2005) conducted the meta-analysis using mixed parameters rather than single parameters in our study. Terrer et al. (2016) assessed the responses of plant biomass using a smaller dataset (27 and 56 observations in AM and ECM plants, respectively) than that in our study (85 and 114 observations in AM and ECM plants, respectively). The larger total plant biomass observed in AM plants (+33.90%) than in ECM plants (+20.57%) in our study may be the result of the larger increase in the net photosynthesis assimilation rate in AM plants (+59.86%) than in ECM plants (+12.50%). This possibility was supported by the higher gross and net plant primary production (GPP and NPP) in AM-dominated ecosystems reported by Averill et al. (2014) and Vargas et al. (2010). Regarding to mycorrhizal fungal growth in our study, ECM fungi demonstrated stronger responses in terms of mycorrhizal fungal biomass (+29.98%) under eCO_2_ compared with AM fungi (+6.61%) (Figure 3). The larger biomass in ECM fungi observed in our study could be explained by the following mechanisms: it is reported that approximately 30% of the total photoassimilation products are used to maintain fungal growth (Nehls and Hampp, 2000), and ECM plants typically allocate more C to their fungal partner than do AM plants (Gehring et al., 2006; Orwin et al., 2011; Soudzilovskaia et al., 2015). Furthermore, one proposed framework postulated that forests dominated by AM trees have an inorganic nutrient economy, whereas forests dominated by ECM trees have an organic nutrient economy (Phillips et al., 2013). The framework in Phillips et al. (2013) speculate that the slow decomposition of litter in these soils results in a greater accumulation of soil organic matter (SOM). Thus, the large proportion of C allocating belowground is used by ECM fungi to acquire N and P from SOM. For different patterns in AM and ECM plants, the larger ECM plant biomass reported in a previous study (Terrer et al., 2016) was explained by the following mechanism: AM trees have a faster leaf litter decomposition rate than ECM trees, this rapid decomposition of AM leaf litter results in the formation of stable mineral-associated organic matter in AM-symbiont soil systems (Sulman et al., 2014; Cotrufo et al., 2015) that cannot be absorbed by AM fungi and plants (Cornelissen et al., 2001; Read and Perez-Moreno, 2003). The above-described evidence indicated that ECM fungi could provide more available N for plants. However, the significantly higher N and P contents in AM plants than in ECM plants obtained in our study may be explained by the possibility that the uptake of plant nutrients might depend heavily on the high C accumulation from photosynthesis under eCO_2_, which was lower in ECM plants than in AM plants. The results in our study corroborate the results described by Phillips et al. (2013), who found a smaller N:C ratio in ECM-dominated plots than in AM-dominated plots. The above data indicated that AM symbionts contributed more to plant growth than ECM symbionts, whereas ECM symbionts contributed more to mycorrhizal fungal growth.

In our study, the responses of mycorrhizal fungi to eCO_2_ differed significantly between the short-term group and the long-term group. However, the responses of mycorrhizal plants to eCO_2_ did not differ significantly between the short-term and long-term groups, indicating a reduced positive effect as the experimental duration was extended. Many mechanisms could explain this positive acclimation effect in plant biomass responses under eCO_2_. Prolonged exposure to eCO_2_ generally reduces the initial stimulation of photosynthesis in many species (Long and Drake, 1991; Ziska et al., 1991) and frequently suppresses photosynthesis (Couture et al., 2014; Kostiainen et al., 2014). These plant responses are further attributed to secondary responses that are related to either excess carbohydrate accumulation or decreased N content rather than directly to eCO_2_ (Chapin et al., 1987; Makino and Mae, 1999). First, increased photosynthesis in response to eCO_2_ could result in excess carbohydrates in plants, as described above, which would subsequently downregulate photosynthesis. Second, as shown in our study, the N in plants was diluted in the absence of an increased nutrient supply in the soil, resulting in decreased photosynthesis and hence a gradual decrease in the beneficial effect. A reduced positive effect might also be found for progressive nitrogen limitation (PNL) in long-term experiments. PNL describes the notion that the stimulation of plant growth by eCO2 results in increased N sequestration in plants, litter and SOM, eventually leading to a progressive decline in soil N availability for plant growth over time (Luo et al., 2004). The reduced N availability, in turn, constrains the eCO_2_ fertilization effect on plant growth over longer timescales, and the mycorrhizal plant partners cause mycorrhizal fungal-induced PNL, reducing the positive effect in plants (Alberton et al., 2007; Liang et al., 2016). A primary mechanism driving this response is the rapid rate of N immobilization by plants and microbes under eCO_2_, which depletes the soil N content and causes slower rates of N mineralization (Finzi et al., 2006). Both AM (Hodge and Fitter, 2010) and ECM (Franklin et al., 2014) fungi can therefore immobilize substantial amounts of N in their tissues, and an increased fungal response to eCO_2_ could result in what has been described as the PNL, as argued by Alberton et al. (2007). The significantly different responses of mycorrhizal fungi to eCO_2_ in the short-term and long-term groups in our study indicate that the PNL has little effect on mycorrhizal fungi.

Larger significant effects on the total plant and shoot biomass were observed in the high N addition experiments in our study. Two mechanisms might explain the improved plant biomass observed with high N addition under eCO_2_ as follows: 1) the addition of N increases the N:C ratio and thereby relieves the negative photosynthesis feedback caused by N limitation, and 2) the increased N input could relieve mycorrhiza-induced PNL (Alberton and Kuyper, 2009) and offset the PNL by increasing the ecosystem N capital under eCO_2_ (Luo et al., 2004). Phillips et al. (2009) reported evidence that the increased allocation of C to root exudates might be a mechanism that delays PNL in forested ecosystems. The lack of a significant difference in the effect sizes of root biomass between low N (+0.340) and high N (+0.275) conditions observed in our study might provide supporting evidence for this hypothesis. One hypothesis assumes that plants would invest more C in mycorrhizal fungi when N or P limited plant growth, since mycorrhizal fungi contribute to the nutrient uptake of plants (Mosse and Phillips, 1971). This report opposite to the idea that the increased N supply might suppress the abundance of mycorrhizal fungi (Treseder, 2004). Plants in nutrient-rich or well-fertilized (high N, high P) soils tend to be less frequently colonized by AM fungi (Staddon et al., 2004), and our study showed that changes in mycorrhizal fungal colonization depend heavily on N sufficiency rather than P sufficiency. Jakobsen et al. (2016) reported that the plant growth responses to elevated atmospheric CO_2_ are increased by a sufficient P supply rather than by arbuscular mycorrhizae. Plant P acquisition is increased by extensive root development and is therefore determined by the C status of plants; notably, the P status influences plant photosynthesis and growth rates, leading to multiple C-P interactions (Jakobsen et al., 2016). Our study showed a slightly higher increase in plant biomass under high P conditions than under low P conditions, and the decreased total plant N:P ratio in our study might indicate potential shifts from P limitation to N limitation in plants. Furthermore, the increased C status of pines under eCO_2_ might facilitate the uptake of limiting P in native ecosystems (Delucia et al., 1997). Integrating nutrient dynamics into terrestrial C cycle models, particularly the limitations on plant growth imposed by N and P availability, has suggested that the land C sink is overestimated in models without these limitations (Wang and Houlton, 2009; Wang et al., 2010; Zhang et al., 2014). A larger increase in the extraradical hyphal length under high P conditions was found in our study, and this finding might be explained by the need for longer extraradical hyphae to obtain more N. While the N addition relieved PNL in plants, larger C increases resulted in positive feedback in mycorrhizal fungi. However, the addition of N to P-rich soils decreased the AM fungal biomass and the mycorrhizal benefits for plant growth (Blanke et al., 2005), whereas N fertilization of P-limited soils increased the fungal biomass and plant growth benefits (Johnson et al., 2003). This finding was consistent with the larger mycorrhizal fungal biomass observed in our study under high N and low P conditions.

The difference between mixtures of different plants and single species of plants had no significant effects on root biomass, indicating that plant species richness did not significantly affect the root biomass under eCO_2_ in our study. Furthermore, a previous study reported that eCO_2_ reduced the loss of plant diversity caused by N deposition (Reich, 2009), and afterward, the whole ecosystem responded to eCO_2_ changes by a feedback effect caused by the plant community shift (Langley and Megonigal, 2010). The mycorrhizal fungal species composition can also be changed by eCO_2_ (Denef et al., 2007; Cotton et al., 2015; Godbold et al., 2015). Several studies have reported that eCO_2_ alters the fungal communities, and positive responses in terms of fungal species richness are rare (Lipson et al., 2014). Our study showed that mixtures of mycorrhizal fungi had greater effects on total plant biomass, root biomass and mycorrhizal fungal colonization under eCO_2_. Rillig et al. (1999) suggested that finding direct relationships between structural data and functional changes is difficult, although a previous study (Klironomos et al., 1998) demonstrated shifts in the mycorrhizal fungal composition. The manner in which functional diversity changes with alterations in the mycorrhizal fungal species richness is not well understood. Klironomos et al. (2005) reported that eCO_2_ leads to a loss of the most C-demanding AM fungi, whereas Kohler et al. (2010) showed decreased fungal species richness unaccompanied by changes in functional diversity. Additional studies should be conducted to assess the functional changes that result from shifts in the mycorrhizal fungal species composition.

In summary, we observed some new findings by our meta-analysis, and the different patterns between AM and ECM symbionts under eCO_2_ in our study could be overall described as follows: the net photosynthesis assimilation rate increased more in AM plants than in ECM plants under eCO_2_, which led to a larger biomass in AM plants than in ECM plants. Subsequently, the total plant nutrient contents (N and P) increased. Larger contents of N in AM plant shoots resulted in positive feedback to the net photosynthesis assimilation rate in the short-term. In addition, lower N:P ratios in AM plants resulted in a larger biomass in AM plants, according to the growth rate hypothesis. However, as the experimental duration increased, the excessive accumulation of photosynthates and the reduced N and P concentrations reduced the positive effect on plant biomass, which in turn resulted in a lower rate for the increase in plant biomass in the long-term experiments. Thus, eCO_2_ strengthened the relationship between mycorrhizal plants and fungi, which led to a greater increase in mycorrhizal fungal biomass, and ECM benefited more from this increase. The exogenous input of N significantly improved plant biomass while inhibiting extraradical hyphae extension.

## Conclusions

Our study demonstrated distinctly different patterns between AM and ECM symbionts under eCO_2_: AM symbionts exhibit greater plant growth, whereas ECM symbionts show greater fungal growth. In addition, the species richness, experimental duration, and fertilization were found to influence the responses of mycorrhizal fungal-plant symbionts to eCO_2_.

Figuring out these different patterns in the responses of mycorrhizal plant-fungal symbionts to eCO_2_ will aid the identification of trends in the development of mycorrhizal plants and fungi under eCO_2_.

## Author contributions

YD, HS, WY, and HX planned and designed the research. YD and HS collected and analyzed the data and wrote the manuscript, and YD and ZW organized the manuscript structure. WY, ZW, and HX polished the English to improve the quality of this manuscript. All authors reviewed and approved the final manuscript.

### Conflict of interest statement

The authors declare that the research was conducted in the absence of any commercial or financial relationships that could be construed as a potential conflict of interest.

## Supplementary material

Table S1 Results of the Egger test for publication bias for 27 variables and of the trim and fill analysis for variables with potential publication bias.

Appendix S1 References used in this meta-analysis.
