# Supplementary material for: The Response Patterns of Arbuscular Mycorrhizal and Ectomycorrhizal Symbionts Under Elevated CO2: A Meta-Analysis
Source: Front Microbiol. 2018 Jun 11;9:1248. doi: 10.3389/fmicb.2018.01248 (PMC6004511; doi:10.3389/fmicb.2018.01248)
Supplement: Table S1 — Results of the Egger test for publication bias for 27 variables and of the trim and fill analysis for variables with potential publication bias. [file Table_1.DOCX]

**Table S1** Results of Egger test for publication bias of 27 variables and trim and fill analysis for variables with potential publication bias.

| variables | Bias p value（P>\|t\|） | Bias 95% CI below | Bias 95% CI up | Trim and fill analysis |
| --- | --- | --- | --- | --- |
| Total plant biomass | 0.000 | -0.737 | -0.226 | No trimming performed; data unchanged |
| Leaf or needle biomass or area | 0.480 | -1.958 | 0.947 |  |
| Shoot biomass | 0.982 | -0.238 | 0.233 |  |
| Root biomass | 0.093 | -0.579 | 0.045 |  |
| Shoot-to-root ratio | 0.000 | 0.802 | 1.552 | No trimming performed; data unchanged |
| N content in total plant | 0.347 | -2.231 | 0.826 |  |
| P content in total plant | 0.407 | -1.510 | 0.660 |  |
| N content in root | 0.896 | -0.939 | 1.066 |  |
| P content in root | 0.829 | -0.654 | 0.532 |  |
| N content in shoot | 0.048 | -2.507 | -0.012 | No trimming performed; data unchanged |
| P content in shoot | 0.035 | -3.469 | -0.151 | Filled 6 results |
| N concentration in total plant | 0.026 | -4.603 | -0.365 | No trimming performed; data unchanged |
| P concentration in total plant | 0.650 | -1.898 | 2.908 |  |
| N concentration in root | 0.404 | -1.279 | 0.531 |  |
| P concentration in root | 0.686 | -0.917 | 1.381 |  |
| N concentration in shoot | 0.613 | -0.634 | 1.060 |  |
| P concentration in shoot | 0.527 | -0.769 | 1.478 |  |
| N concentration in leaf | 0.276 | -2.914 | 0.891 |  |
| P concentration in leaf | 0.013 | -1.845 | -0.233 | Filled 3 results |
| Net photosynthesis assimilation rate | 0.704 | -2.684 | 3.868 | No trimming performed; data unchanged |
| TSS concentration in leaf | 0.022 | -2.962 | -0.265 | No trimming performed; data unchanged |
| Mycorrhizal fungal colonization | 0.004 | 0.105 | 0.547 | No trimming performed; data unchanged |
| Root with hyphae | 0.095 | -0.069 | 0.839 |  |
| Root with arbuscules | 0.000 | 0.583 | 1.633 | Filled 2 results |
| Root with vesicles | 0.053 | -0.013 | 2.053 |  |
| Extraradical hyphal length | 0.987 | -0.349 | 0.354 |  |
| Mycorrhizal fungal biomass | 0.997 | -0.407 | 0.408 |  |
