# Supplementary material for: The Response Patterns of Arbuscular Mycorrhizal and Ectomycorrhizal Symbionts Under Elevated CO2: A Meta-Analysis
Source: Front Microbiol. 2018 Jun 11;9:1248. doi: 10.3389/fmicb.2018.01248 (PMC6004511; doi:10.3389/fmicb.2018.01248)
Supplement: Appendix S1 — References used in this meta-analysis. [file Data_Sheet_1.docx]

**Appendix S1** References on this meta-analysis

1. Alberton, O., Kuyper, T. W. & Gorissen, A. Competition for nitrogen between *Pinus sylvestris* and ectomycorrhizal fungi generates potential for negative feedback under elevated CO2. *Plant and Soil* **296**, 159-172 (2007).
2. Alberton, O. & Kuyper, T. W. Ectomycorrhizal fungi associated with *Pinus sylvestris* seedlings respond differently to increased carbon and nitrogen availability: implications for ecosystem responses to global change. *Global Change Biology* **15**, 166-175 (2009).
3. Andersen, F. Ø. & Andersen, T. Effects of arbuscular mycorrhizae on biomass and nutrients in the aquatic plant *Littorella uniflora*. *Freshwater Biology* **51**, 1623-1633 (2006).
4. Arndal, M. F. *et al.* Net root growth and nutrient acquisition in response to predicted climate change in two contrasting heathland species. *Plant and Soil* **369**, 615-629 (2013).
5. Baslam, M., Antolín, M. C., Gogorcena, Y., Muñoz, F. & Goicoechea, N. Changes in alfalfa forage quality and stem carbohydrates induced by arbuscular mycorrhizal fungi and elevated atmospheric CO2. *Annals of applied biology* **164**, 190-199 (2014).
6. Baslam, M., Erice, G. & Goicoechea, N. Impact of arbuscular mycorrhizal fungi (AMF) and atmospheric CO2 concentration on the biomass production and partitioning in the forage legume alfalfa. *Symbiosis* **58**, 171-181 (2012).
7. Baslam, M., Garmendia, I. & Goicoechea, N. Elevated CO2 may impair the beneficial effect of arbuscular mycorrhizal fungi on the mineral and phytochemical quality of lettuce. *Annals of Applied Biology* **161**, 180-191 (2012).
8. Becklin, K. M., Mullinix, G. W. & Ward, J. K. Host plant physiology and mycorrhizal functioning shift across a glacial through future CO2 gradient. *Plant physiology* **172**, 789-801 (2016).
9. Bettoni, M. M., Mogor, Á. F., Pauletti, V. & Goicoechea, N. Growth and metabolism of onion seedlings as affected by the application of humic substances, mycorrhizal inoculation and elevated CO2. *Scientia Horticulturae* **180**, 227-235 (2014).
10. Berntson, G. M. & Bazzaz, F. A. Regenerating temperate forest mesocosms in elevated CO2: belowground growth and nitrogen cycling. *Oecologia* **113**, 115-125 (1997).
11. Berntson, G. M., Wayne, P. M. & Bazzaz, F. A. Below-ground architectural and mycorrhizal responses to elevated CO2 in *Betula alleghaniensis* populations. *Functional Ecology* **11**, 684-695 (1997).
12. Carrillo, Y., Dijkstra, F. A., LeCain, D. & Pendall, E. Mediation of soil C decomposition by arbuscular mycorrizhal fungi in grass rhizospheres under elevated CO2. *Biogeochemistry* **127**, 45-55 (2016).
13. Cavagnaro, T. R., Sokolow, S. K. & Jackson, L. E. Mycorrhizal effects on growth and nutrition of tomato under elevated atmospheric carbon dioxide. *Functional Plant Biology* **34**, 730-736 (2007).
14. Chen, X. *et al.* Plant nitrogen acquisition and interactions under elevated carbon dioxide: impact of endophytes and mycorrhizae. *Global Change Biology* **13**, 1238-1249 (2007).
15. Choi, D. S., Quoreshi, A. M., Maruyama, Y., Jin, H. O. & Koike, T. Effect of ectomycorrhizal infection on growth and photosynthetic characteristics of *Pinus densiflora* seedlings grown under elevated CO_2_ concentrations. *Photosynthetica* **43**, 223-229 (2005).
16. Choi, D. S., Makoto, K., Quoreshi, A. M., Qu, L. Seed germination and seedling physiology of *Larix kaempferi* and *Pinus densiflora* in seedbeds with charcoal and elevated CO2. *Landscape and Ecological Engineering* **5**, 107-113 (2009).
17. Choi, D. S., Kayama, M., Chung, D. J., Jin, H. O., Quoreshi, A. M., Maruyama, Y., Koike, T. 2005a. Mycorrhizal activities in *Pinus densiflora*, *P. koraiensis* and *Larix kaempferi* native to Korea raised under high CO2 concentrations and water use efficiency. *Phyton* **45**, 139-144 (2005a).
18. Clark, N. M., Rillig, M. C. & Nowak, R. S. Arbuscular mycorrhizal fungal abundance in the Mojave Desert: seasonal dynamics and impacts of elevated CO_2_. *Journal of Arid Environments* **73**, 834-843 (2009).
19. Constable, J. V., Bassirirad, H., Lussenhop, J. & Zerihun, A. Influence of elevated CO_2_ and mycorrhizae on nitrogen acquisition: contrasting responses in *Pinus taeda* and *Liquidambar styraciflua*. *Tree Physiology* **21**, 83-91 (2001).
20. del Mar Alguacil, M., Kohler, J., Caravaca, F. & Roldán, A. Differential effects of *Pseudomonas mendocina* and *Glomus intraradices* on lettuce plants physiological response and aquaporin PIP2 gene expression under elevated atmospheric CO_2_ and drought. *Microbial ecology* **58**, 942-951 (2009).
21. Delucia, E. H., Callaway, R. M., Thomas, E. M. & Schlesinger, W. H. Mechanisms of phosphorus acquisition for ponderosa pine seedlings under high CO_2_ and temperature. *Annals of Botany* **79**, 111-120 (1997).
22. Denef, K. *et al.* Community shifts and carbon translocation within metabolically-active rhizosphere microorganisms in grasslands under elevated CO_2_. *Biogeosciences* **4**, 769-779 (2007).
23. Dhillion, S. S., Roy, J. & Abrams, M. Assessing the impact of elevated CO_2_ on soil microbial activity in a Mediterranean model ecosystem. *Plant and soil* **187**, 333-342 (1995).
24. Fabbrin, E. G., Gogorcena, Y., Mogor, Á. F., Garmendia, I. & Goicoechea, N. Pearl millet growth and biochemical alterations determined by mycorrhizal inoculation, water availability and atmospheric CO_2_ concentration. *Crop and Pasture Science* **66**, 831-840 (2015).
25. Fortuna, P., Avio, L., Morini, S. & Giovannetti, M. Fungal biomass production in response to elevated atmospheric CO_2_ in a *Glomus mosseae*-*Prunus cerasifera* model system. *Mycological progress* **11**, 17-26 (2012).
26. Fransson, P. M., Taylor, A. F. & Finlay, R. D. Mycelial production, spread and root colonisation by the ectomycorrhizal fungi *Hebeloma crustuliniforme* and *Paxillus involutus* under elevated atmospheric CO_2_. *Mycorrhiza* **15**, 25-31 (2005).
27. Fransson, P. M. A., Anderson, I. C. & Alexander, I. J. Does carbon partitioning in ectomycorrhizal pine seedlings under elevated CO_2_ vary with fungal species? *Plant and Soil* **291**, 323-333 (2007).
28. Fransson, P. M. A. & Johansson, E. M. Elevated CO_2_ and nitrogen influence exudation of soluble organic compounds by ectomycorrhizal root systems. *FEMS microbiology ecology* **71**, 186-196 (2010).
29. Gamper, H., Hartwig, U. A. & Leuchtmann, A. Mycorrhizas improve nitrogen nutrition of *Trifolium repens* after 8 yr of selection under elevated atmospheric CO_2_ partial pressure. *New Phytologist* **167**, 531-542 (2005).
30. Gamper, H. *et al.* Arbuscular mycorrhizal fungi benefit from 7 years of free air CO_2_ enrichment in well-fertilized grass and legume monocultures. *Global Change Biology* **10**, 189-199 (2004).
31. Garcia, M. O., Ovasapyan, T., Greas, M. & Treseder, K. K. Mycorrhizal dynamics under elevated CO_2_ and nitrogen fertilization in a warm temperate forest. *Plant and Soil* **303**, 301-310 (2008).
32. Gavito, M. E., Curtis, P. S., Mikkelsen, T. N. & Jakobsen, I. Atmospheric CO_2_ and mycorrhiza effects on biomass allocation and nutrient uptake of nodulated pea (*Pisum sativum* L.) plants. *Journal of Experimental Botany* **51**, 1931-1938 (2000).
33. Gavito, M. E., Bruhn, D. & Jakobsen, I. Phosphorus uptake by arbuscular mycorrhizal hyphae does not increase when the host plant grows under atmospheric CO_2_ enrichment. *New Phytologist* **154**, 751-760 (2002).
34. Gavito, M. E., Schweiger, P. & Jakobsen, I. P uptake by arbuscular mycorrhizal hyphae: effect of soil temperature and atmospheric CO_2_ enrichment. *Global Change Biology* **9**, 106-116 (2003).
35. Godbold, D. L., Berntson, G. M. & Bazzaz, F. Growth and mycorrhizal colonization of three North American tree species under elevated atmospheric CO_2_. *New Phytologist* **137**, 433-440 (1997).
36. Goicoechea, N., Bettoni, M., Fuertes-Mendizábal, T., González-Murua, C. & Aranjuelo, I. Durum wheat quality traits affected by mycorrhizal inoculation, water availability and atmospheric CO_2_ concentration. *Crop and Pasture Science* **67**, 147-155 (2016).
37. Gutknecht, J. L., Field, C. B. & Balser, T. C. Microbial communities and their responses to simulated global change fluctuate greatly over multiple years. *Global Change Biology* **18**, 2256-2269 (2012).
38. Hu, S., Wu, J., Burkey, K. O. & Firestone, M. K. Plant and microbial N acquisition under elevated atmospheric CO_2_ in two mesocosm experiments with annual grasses. *Global Change Biology* **11**, 213-223 (2005).
39. Humphreys, C. P. *et al.* Mutualistic mycorrhiza-like symbiosis in the most ancient group of land plants. *Nature communications* **1**, 103 (2010).
40. Ineichen, K., Wiemken, V. & Wiemken, A. Shoots, roots and ectomycorrhiza formation of pine seedlings at elevated atmospheric carbon dioxide. *Plant, Cell & Environment* **18**, 703-707 (1995).
41. Izumi, H., Elfstrand, M. & Fransson, P. *Suillus* mycelia under elevated atmospheric CO_2_ support increased bacterial communities and scarce *nifH* gene activity in contrast to *Hebeloma* mycelia. *Mycorrhiza* **23**, 155-165 (2013).
42. Jifon, J. L., Graham, J. H., Drouillard, D. L. & Syvertsen, J. P. Growth depression of mycorrhizal *Citrus* seedlings grown at high phosphorus supply is mitigated by elevated CO_2_. *New Phytologist* **153**, 133-142 (2002).
43. Johansson, E. M., Fransson, P. M. A., Finlay, R. D. & van Hees, P. A. W. Quantitative analysis of soluble exudates produced by ectomycorrhizal roots as a response to ambient and elevated CO_2_. *Soil Biology and Biochemistry* **41**, 1111-1116 (2009).
44. Johnson, N. C., Wolf, J. & Koch, G. W. Interactions among mycorrhizae, atmospheric CO_2_ and soil N impact plant community composition. *Ecology Letters* **6**, 532-540 (2003).
45. Jongen, M., Fay, P. & Jones, M. B. Effects of elevated carbon dioxide and arbuscular mycorrhizal infection on *Trifolium repens*. *New Phytologist* **132**, 413-423 (1996).
46. Kasurinen, A., Helmisaari, H.-S. & Holopainen, T. The influence of elevated CO_2_ and O_3_ on fine roots and mycorrhizas of naturally growing young Scots pine trees during three exposure years. *Global Change Biology* **5**, 771-780 (1999).
47. Klironomos, J., Rillig, M. & Allen, M. Below-ground microbial and microfaunal responses to *Artemisia tridentata* grown under elevated atmospheric CO_2_. *Functional Ecology*, 527-534 (1996).
48. Klironomos, J. *et al.* Soil fungal-arthropod responses to *Populus tremuloides* grown under enriched atmospheric CO_2_ under field conditions. *Global Change Biology* **3**, 473-478 (1997).
49. Klironomos, J. N., Ursic, M., Rillig, M. & Allen, M. F. Interspecific differences in the response of arbuscular mycorrhizal fungi to *Artemisia tridentata* grown under elevated atmospheric CO_2_. *New Phytologist* **138**, 599-605 (1998).
50. Klironomos, J. N. *et al.* Abrupt rise in atmospheric CO_2_ overestimates community response in a model plant-soil system. *Nature* **433**, 621-624 (2005).
51. Kogawara, S., Norisada, M., Tange, T., Yagi, H. & Kojima, K. Elevated atmospheric CO_2_ concentration alters the effect of phosphate supply on growth of Japanese red pine (*Pinus densiflora*) seedlings. *Tree Physiology* **26**, 25-33 (2006).
52. Kohler, J., Caravaca, F., del Mar Alguacil, M. & Roldán, A. Elevated CO_2_ increases the effect of an arbuscular mycorrhizal fungus and a plant-growth-promoting rhizobacterium on structural stability of a semiarid agricultural soil under drought conditions. *Soil Biology and Biochemistry* **41**, 1710-1716 (2009).
53. Kytöviita, M. M., Pelloux, J., Fontaine, V., Botton, B. & Dizengremel, P. Elevated CO_2_ does not ameliorate effects of ozone on carbon allocation in *Pinus halepensis* and *Betula pendula* in symbiosis with *Paxillus involutus*. *Physiologia Plantarum* **106**, 370-377 (1999).
54. Kytöviita, M.-M., Le Thiec, D. & Dizengremel, P. Elevated CO_2_ and ozone reduce nitrogen acquisition by *Pinus halepensis* from its mycorrhizal symbiont. *Physiologia Plantarum* **111**, 305-312 (2001).
55. Langley, A. J., Dijkstra, P., Drake, G. B. & Hungate, A. B. Ectomycorrhizal colonization, biomass, and production in a regenerating scrub oak forest in response to elevated CO_2_. *Ecosystems* **6**, 424-430 (2003).
56. Lewis, J. D., Thomas, R. B. & Strain, B. R. Effect of elevated CO_2_ on mycorrhizal colonization of loblolly pine (*Pinus taeda* L.) seedlings. *Plant and Soil* **165**, 81-88 (1994).
57. Lewis, J. D. & Strain, B. R. The role of mycorrhizas in the response of *Pinus taeda* seedlings to elevated CO_2_. *New Phytologist* **133**, 431-443 (1996).
58. Loewe, A., Einig, W., Shi, L., Dizengremel, P. & Hampp, R. Mycorrhiza formation and elevated CO_2_ both increase the capacity for sucrose synthesis in source leaves of spruce and aspen. *New Phytologist* **145**, 565-574 (2000).
59. Louche-Tessandier D., Samson G., Hernandez-Sebastia C., Chagvardieff, P. & Desjardins, Y. Importance of light and CO_2_ on the effects of endomycorrhizal colonization on growth and photosynthesis of potato plantlets (*Solanum tuberosum*) in an in vitro tripartite system. *New Phytologist* **142**, 539-550 (1999).
60. Lovelock, C., Kyllo, D. & Winter, K. Growth responses to vesicular-arbuscular mycorrhizae and elevated CO_2_ in seedlings of a tropical tree, *Beilschmiedia pendula*. *Functional Ecology*, 662-667 (1996).
61. Lovelock, C. *et al.* Symbiotic vesicular-arbuscular mycorrhizae influence maximum rates of photosynthesis in tropical tree seedlings grown under elevated CO_2_. *Functional Plant Biology* **24**, 185-194 (1997).
62. Lukac, M., Calfapietra, C. & Godbold, D. L. Production, turnover and mycorrhizal colonization of root systems of three *Populus* species grown under elevated CO_2_ (POPFACE). *Global Change Biology* **9**, 838-848 (2003).
63. Lussenhop, J., Treonis, A., Curtis, P. S., Teeri, J. A. & Vogel, C. S. Response of soil biota to elevated atmospheric CO_2_ in poplar model systems. *Oecologia* **113**, 247-251 (1998).
64. Markkola *et al.* Scots pine responses to CO_2_ enrichment. I. Ectomycorrhizal fungi and soil fauna. *Environmental Pollution* **94**, 309-316 (1996).
65. Norisada, M., Motoshige, T., Kojima, K. & Tange, T. Effects of phosphate supply and elevated CO_2_ on root acid phosphatase activity in *Pinus densiflora* seedlings. *Journal of Plant Nutrition and Soil Science* **169**, 274-279 (2006).
66. Olesniewicz, K. S. & Thomas, R. B. Effects of mycorrhizal colonization on biomass production and nitrogen fixation of black locust (*Robinia pseudoacacia*) seedlings grown under elevated atmospheric carbon dioxide. *New Phytologist* **142**, 133-140 (1999).
67. O'Neill, E. G., Luxmoore, R. J. & Norby, R. J. Increases in mycorrhizal colonization and seedling growth in *Pinus echinata* and *Quercus alba* in an enriched CO_2_ atmosphere. *Canadian Journal of Forest Research* **17**, 878-883 (1987).
68. Pérez-Soba, M., Dueck, T. A., Puppi, G. & Kuiper, P. J. C. Interactions of elevated CO_2_, NH_3_ and O_3_ on mycorrhizal infection, gas exchange and N metabolism in saplings of Scots pine. *Plant and Soil* **176**, 107-116 (1995).
69. Plett, J. M. *et al.* The effect of elevated carbon dioxide on the interaction between *Eucalyptus grandis* and diverse isolates of *Pisolithus sp.* is associated with a complex shift in the root transcriptome. *New Phytologist* **206**, 1423-1436 (2015).
70. Rillig, M. C., Allen, M. F., Klironomos, J. N., Chiariello, N. R. & Field, C. B. Plant species-specific changes in root-inhabiting fungi in a California annual grassland: responses to elevated CO_2_ and nutrients. *Oecologia* **113**, 252-259 (1998).
71. Rillig, M. C., Allen, M. F., Klironomos, J. N. & Field, C. B. Arbuscular mycorrhizal percent root infection and infection intensity of *Bromus hordeaceus* grown in elevated atmospheric CO₂. *Mycologia*, 199-205 (1998).
72. Rillig, M. C., Field, C. B. & Allen, M. F. Soil biota responses to long-term atmospheric CO_2_ enrichment in two California annual grasslands. *Oecologia* **119**, 572-577 (1999).
73. Rillig, M. C., Field, C. B. & Allen, M. F. Fungal root colonization responses in natural grasslands after long-term exposure to elevated atmospheric CO_2_. *Global Change Biology* **5**, 577-585 (1999).
74. Rey, A., Barton, C. V. M. & Jarvis, P. G. in *Impacts of Global Change on Tree Physiology and Forest Ecosystems* (eds G. M. J. Mohren, K. Kramer, & S. Sabaté) 207-211 (Springer Netherlands, Dordrecht, 1997).
75. Rillig, M. C. & Field, C. B. Arbuscular mycorrhizae respond to plants exposed to elevated atmospheric CO_2_ as a function of soil depth. *Plant and Soil* **254**, 383-391 (2003).
76. Rønn, R. *et al.* Response of free-living soil protozoa and microorganisms to elevated atmospheric CO_2_ and presence of mycorrhiza. *Soil Biology and Biochemistry* **34**, 923-932 (2002).
77. Rouhier, H. & Read, D. J. The role of mycorrhiza in determining the response of *Plantago lanceolata* to CO_2_ enrichment. *New Phytologist* **139**, 367-373 (1998).
78. Rouhier, H. & Read, D. J. Plant and fungal responses to elevated atmospheric carbon dioxide in mycorrhizal seedlings of *Pinus sylvestris*. *Environmental and Experimental Botany* **40**, 237-246 (1998).
79. Rouhier, H. & Read, D. J. Plant and fungal responses to elevated atmospheric CO_2_ in mycorrhizal seedlings of *Betula pendula*. *Environmental and Experimental Botany* **42**, 231-241 (1999).
80. Rúa, M. A., Umbanhowar, J., Hu, S., Burkey, K. O. & Mitchell, C. E. Elevated CO_2_ spurs reciprocal positive effects between a plant virus and an arbuscular mycorrhizal fungus. *New Phytologist* **199**, 541-549 (2013).
81. Runion, G. B. *et al.* Effects of free-air CO_2_ enrichment on microbial populations in the rhizosphere and phyllosphere of cotton. *Agricultural and Forest Meteorology* **70**, 117-130 (1994).
82. Sanders, I., Streitwolf-Engel, R., Van der Heijden, M., Boller, T. & Wiemken, A. Increased allocation to external hyphae of arbuscular mycorrhizal fungi under CO_2_ enrichment. *Oecologia* **117**, 496-503 (1998).
83. Seegmüller, S. & Rennenberg, H. Interactive effects of mycorrhization and elevated carbon dioxide on growth of young pedunculate oak (*Quercus robur* L.) trees. *Plant and Soil* **167**, 325-329 (1994).
84. Shinano, T. *et al.* Effects of elevated atmospheric CO_2_ concentration on the nutrient uptake characteristics of Japanese larch (*Larix kaempferi*). *Tree Physiology* **27**, 97-104 (2007).
85. Staddon, P. L., Graves, J. D. & Fitter, A. H. Effect of enhanced atmospheric CO_2_ on mycorrhizal colonization by *Glomus mosseae* in *Plantago lanceolata* and *Trifolium repens*. *New Phytologist* **139**, 571-580 (1998).
86. Staddon, P. L., Fitter, A. H. & Graves, J. D. Effect of elevated atmospheric CO_2_ on mycorrhizal colonization, external mycorrhizal hyphal production and phosphorus inflow in *Plantago lanceolata* and *Trifolium repens* in association with the arbuscular mycorrhizal fungus *Glomus mosseae*. *Global Change Biology* **5**, 347-358 (1999).
87. Staddon, P., Fitter, A. & Robinson, D. Effects of mycorrhizal colonization and elevated atmospheric carbon dioxide on carbon fixation and below-ground carbon partitioning in *Plantago lanceolata*. *Journal of Experimental Botany* **50**, 853-860 (1999).
88. Staddon, P. L., Gregersen, R. & Jakobsen, I. The response of two Glomus mycorrhizal fungi and a fine endophyte to elevated atmospheric CO_2_, soil warming and drought. *Global Change Biology* **10**, 1909-1921 (2004).
89. Staddon, P. L., Jakobsen, I. & Blum, H. Nitrogen input mediates the effect of free-air CO_2_ enrichment on mycorrhizal fungal abundance. *Global Change Biology* **10**, 1678-1688 (2004).
90. Staddon, P. L. *et al.* A decade of free-air CO_2_ enrichment increased the carbon throughput in a grass-clover ecosystem but did not drastically change carbon allocation patterns. *Functional ecology* **28**, 538-545 (2014).
91. Tang, J., Chen, J. & Chen, X. Response of 12 weedy species to elevated CO_2_ in low-phosphorus-availability soil. *Ecological Research* **21**, 664-670 (2006).
92. Tang, J., Xu, L., Chen, X. & Hu, S. Interaction between C_4_ barnyard grass and C_3_ upland rice under elevated CO_2_: impact of mycorrhizae. *acta oecologica* **35**, 227-235 (2009).
93. Taylor, B. N. *et al.* Root length, biomass, tissue chemistry and mycorrhizal colonization following 14 years of CO_2_ enrichment and 6 years of N fertilization in a warm temperate forest. *Tree Physiology* **34**, 955-965 (2014).
94. Treseder, K. K., Egerton-Warburton, L. M., Allen, M. F., Cheng, Y. & Oechel, W. C. Alteration of soil carbon pools and communities of mycorrhizal fungi in chaparral exposed to elevated carbon dioxide. *Ecosystems* **6**, 786-796 (2003).
95. Tarnau, K. *et al.* Carbon dioxide concentration and nitrogen input affect the C and N storage pools in *Amanita muscaria*-*Picea abies* mycorrhizae. *Tree Physiology* **21**, 93-99 (2001).
96. Vose, J. M. *et al.* Effects of elevated CO_2_ and N fertilization on soil respiration from ponderosa pine (*Pinus ponderosa*) in open-top chambers. *Canadian Journal of Forest Research* **25**, 1243-1251 (1995).
97. Walker, R. F., Geisinger, D. R., Johnson, D. W. & Ball, J. T. Enriched atmospheric CO_2_ and soil P effects on growth and ectomycorrhizal colonization of juvenile ponderosa pine. *Forest Ecology and Management* **78**, 207-215 (1995).
98. Walker, R. F., Geisinger, D. R., Johnson, D. W. & Ball, J. T. Elevated atmospheric CO_2_ and soil N fertility effects on growth, mycorrhizal colonization, and xylem water potential of juvenile ponderosa pine in a field soil. *Plant and Soil* **195**, 25-36 (1997).
99. Walker, R. F., Geisinger, D. R., Johnson, D. W. & Ball, J. T. Atmospheric CO_2_ enrichment and soil N fertility effects on juvenile ponderosa pine: Growth, ectomycorrhizal development, and xylem water potential. *Forest Ecology and Management* **102**, 33-44 (1998).
100. Walker, R. F., Johnson, D. W., Geisinger, D. R. & Ball, J. T. Growth and ectomycorrhizal colonization of ponderosa pine seedlings supplied different levels of atmospheric CO_2_ and soil N and P. *Forest Ecology and Management* **109**, 9-20, (1998).
101. Wang, X., Li, S., Bai, K. & Kuang, T. Influences of double aerial CO_2_ concentration on plant root surface area and viability and infection intensity of vesicular-arbuscular mycorrhizal fungi. *Chinese Science Bulletin* **44**, 63-64 (1999).
102. Wang, X. *et al.* Ectomycorrhizal colonization and growth of the hybrid larch F1 under elevated CO_2_ and O_3_. *Environmental Pollution* **197**, 116-126 (2015).
103. Weight, B. R. *et al.* Effects of twice-ambient carbon dioxide and nitrogen amendment on biomass, nutrient contents and carbon costs of *Norway spruce* seedlings as influenced by mycorrhization with *Piloderma croceum* and *Tomentellopsis submollis*. *Mycorrhiza* **21**, 375-391 (2011).
104. Wiemken, V., Ineichen, K. & Boller, T. Development of ectomycorrhizas in model beech-spruce ecosystems on siliceous and calcareous soil: a 4-year experiment with atmospheric CO_2_ enrichment and nitrogen fertilization. *Plant and Soil* **234**, 99-108 (2001).
105. Zhu, X., Song, F., Liu, S. & Liu, F. Arbuscular mycorrhiza improve growth, nitrogen uptake, and nitrogen use efficiency in wheat grown under elevated CO_2_. *Mycorrhiza* **26**, 133-140 (2016).
106. Goicoechea, N., Baslam, M., Erice, G. & Irigoyen, J. J. Increased photosynthetic acclimation in alfalfa associated with arbuscular mycorrhizal fungi (AMF) and cultivated in greenhouse under elevated CO_2_. *Journal of Plant Physiology* **171**, 1774-1781 (2014).
107. Gorissen, A. & Kuyper, T. W. Fungal species-specific responses of ectomycorrhizal Scots pine (*Pinus sylvestris*) to elevated CO_2_. *New Phytologist* **146**, 163-168 (2000).
108. Hartwig, U. A. *et al.* Arbuscular mycorrhiza infection enhances the growth response of *Lolium perenne* to elevated atmospheric pCO_2_. *J Exp Bot* **53**, 1207-1213 (2002).
109. Nowak, J. & Nowak, J. S. CO_2_ enrichment and mycorrhizal effects on cutting growth and some physiological traits of cuttings during rooting. *Acta Scientiarum Polonorum. Hortorum Cultus* **12** (2013).
110. Syvertsen, J. P. & Graham, J. H. Phosphorus supply and arbuscular mycorrhizas increase growth and net gas exchange responses of two *Citrus spp.* grown at elevated CO_2_. *Plant and Soil* **208**, 209-219 (1999).
111. Jakobsen, I. *et al.* Plant growth responses to elevated atmospheric CO_2_ are increased by phosphorus sufficiency but not by arbuscular mycorrhizas. *Journal of Experimental Botany* **67**, 6173-6186 (2016).
112. Zhu, X., Song, F., Liu, S. & Liu, F. Role of arbuscular mycorrhiza in alleviating salinity stress in wheat (*Triticum aestivum* L.) grown under ambient and elevated CO_2_. *Journal of Agronomy and Crop Science* **202**, 486-496 (2016).
